# Is Acculturation Related to Obesity in Hispanic/Latino Adults? Results from the Hispanic Community Health Study/Study of Latinos

**DOI:** 10.1155/2015/186276

**Published:** 2015-03-29

**Authors:** Carmen R. Isasi, Guadalupe X. Ayala, Daniela Sotres-Alvarez, Hala Madanat, Frank Penedo, Catherine M. Loria, John P. Elder, Martha L. Daviglus, Janice Barnhart, Anna Maria Siega-Riz, Linda Van Horn, Neil Schneiderman

**Affiliations:** ^1^Department of Epidemiology & Population Health, Albert Einstein College of Medicine, Bronx, NY 10461, USA; ^2^Division of Health Promotion and Behavioral Science, Graduate School of Public Health, San Diego State University and the Institute for Behavioral and Community Health, San Diego State University Research Foundation, San Diego, CA 92123, USA; ^3^Collaborative Studies Coordinating Center, Department of Biostatistics, Gillings School of Global Public Health, University of North Carolina at Chapel Hill, Chapel Hill, NC 27514, USA; ^4^Department of Medical Social Sciences, Feinberg School of Medicine, Northwestern University, Chicago, IL 60611, USA; ^5^Division of Cardiovascular Sciences, National Heart, Lung, and Blood Institute, Bethesda, MD 20817, USA; ^6^Institute for Minority Health Research, University of Illinois at Chicago, Chicago, IL 60612, USA; ^7^Departments of Epidemiology and Nutrition, Gillings School of Global Public Health, University of North Carolina at Chapel Hill, Chapel Hill, NC 27599, USA; ^8^Department of Preventive Medicine, Feinberg School of Medicine, Northwestern University, Chicago, IL 60126, USA; ^9^The Behavioral Research Center, Department of Psychology, University of Miami, Coral Gables, FL 33124, USA

## Abstract

*Background.* The study examined the association of obesity with acculturation in a large and diverse sample of US Hispanic/Latino adults.* Methods.* The Hispanic Community Health Study (HCHS)/Study of Latinos (SOL) is a community-based cohort study of Hispanic/Latino adults aged 18–74 years (*N* = 16,415) from four urban areas. Height and weight were directly measured using a standardized protocol. Acculturation was assessed by the Short Acculturation Scale for Hispanics (SASH). Other immigration related variables included place of birth, length of residency in the US, and age at immigration. Odds ratios were calculated to assess the association of overweight, moderate obesity, and extreme obesity (≥40 kg/m^2^) with acculturation and sociodemographic variables.* Results.* The prevalence of obesity was 42.4% for women and 36.5% for men and varied by field center and Hispanic/Latino background. The strongest predictor of moderate and extreme obesity was length of residency in mainland US. This association was consistent across Hispanic/Latino backgrounds. Acculturation was not significantly associated with obesity.* Discussion.* The burden of obesity is high among Hispanic/Latino adults. The study findings suggest that prolonged exposure to the environments in these communities, rather than acculturation, is an important risk factor for obesity in this population.

## 1. Introduction

Hispanics living in the US are disproportionately represented in the obesity epidemic. Data from National Health and Nutrition Examination Survey (NHANES) 2009-2010 showed that the prevalence of obesity among Hispanic adults, primarily Mexican Americans, is higher than non-Hispanic whites, with significant increases over the last decade for Mexican Americans [1]. Despite their diversity in origins and culture [2], national survey data do not present a clear picture of the burden of obesity and its risk factors among the various Hispanic groups other than those of Mexican origin. Emerging evidence suggests that country of origin may have important implications for understanding disease risks [3]. In addition, research has shown that being born in the US and longer duration of residency are associated with a higher prevalence of obesity [4], suggesting that acculturation may play a role in the development of obesity. Acculturation research examines the extent to which continuous and first-hand contact with a new dominant culture that is different from one's culture of origin is associated with individual behavior change [5, 6]. However, few studies have examined the association of acculturation with obesity in a diverse sample of Hispanics from different backgrounds or nationalities.

In this paper we examined the association between acculturation and obesity in the largest study of Hispanic/Latino adults living in the US. We also examined differences in the prevalence of obesity by Hispanic/Latino background and socioeconomic status indicators. Furthermore, because the prevalence of extreme obesity has risen in the US, particularly among minority groups [7], and because individuals with extreme obesity have higher CVD risk and mortality [8], we also examined the burden of extreme obesity and the sociodemographic risk factors associated with the prevalence of this condition in this diverse sample of Hispanic/Latino adults.

## 2. Methods

### 2.1. Participants

HCHS/SOL is a community based cohort study of 16,415 self-identified Hispano/Latino individuals aged 18–74 recruited from 2008 to 2011 in four US field centers (Chicago, IL; Miami, FL; Bronx, NY; San Diego, CA). HCHS/SOL participants include those who self-identified their background as Central American (*n* = 1,732), Cuban (*n* = 2,348), Dominican (*n* = 1,473), Mexican (*n* = 6,472), Puerto Rican (*n* = 2,728), and South American (*n* = 1,072). HCHS/SOL goals are to describe the prevalence of risk and protective factors for various chronic conditions (e.g., cardiovascular disease (CVD), diabetes, and pulmonary disease) and to quantify all-cause mortality, fatal and nonfatal CVD and pulmonary disease, and pulmonary disease exacerbation over time [9].

The sample design and cohort selection have been previously described [10]. Briefly, a stratified two-stage area probability sample of household addresses was selected in each of the four field centers. The HCHS/SOL target population is defined as all noninstitutionalized Hispanic/Latino adults aged 18–74 years and residing in the defined geographical areas (census block groups) across the four participating field centers. HCHS/SOL participants were selected using a probability sampling design within these areas to provide a representative sample of the target population. The study oversampled 45–74-year-olds to ensure that the sample size was large enough for analysis of cardiovascular events. Because participants were selected with unequal probabilities, sampling weights were generated to reflect the probabilities of selection at each stage. The study was approved by each field center's institutional review board.

Of the 16,415 participants enrolled in the study, 149 participants were excluded from the analysis because of missing Hispanic/Latino background (*n* = 87), BMI (*n* = 53), or age ineligible (>74 years) at baseline (*n* = 9), leaving an analytic sample of 16,266 participants.

### 2.2. Study Measures

#### 2.2.1. Overweight and Obesity

Height and weight were obtained at each field center clinic using standardized protocols. Height was measured with a wall stadiometer (SECA 222, Germany) and weight was obtained with a digital scale (Tanita Body Composition Analyzer, TBF 300, Japan). Body mass index was calculated as weight in kilograms divided by height in meters squared. Overweight was defined as BMI 25.0 to 29.9 kg/m^2^ and obesity as a BMI of 30.0 kg/m^2^ or higher. Using NHLBI guidelines obese participants were further classified as having moderate obesity (obesity classes I and II, BMI 30.0 to 39.9 kg/m^2^) or extreme obesity (obesity class III, BMI ≥ 40 kg/m^2^) [11].

#### 2.2.2. Measures of Immigration History

Based on participants' place of birth report, they were classified as either born or not outside the US mainland (50 states). Hence, those born in Puerto Rico or other US territories were also classified as born outside US mainland to better reflect their migration and acculturation patterns. Foreign born individuals were asked the number of years lived in the mainland US. Age at immigration was calculated from their reported age and the duration of residence (years) in mainland US.

#### 2.2.3. Measures of Acculturation

Two indices of acculturation were considered. Participants completed an abbreviated 10-item version of Marin's Short Acculturation Scale for Hispanics (SASH) [12], with items related to language preference, media preference, and social affiliations. Two factors were identified (SASH language use, *α* = 0.92, and SASH ethnic social relations, *α* = 0.73), and these two subscales were analyzed separately in the models. Dietary acculturation asked if the types of foods participants usually ate were from Hispanic/Latino or American origin on a 5-level Likert scale ranging from “1 = mostly Hispanic” to “5 = mostly American foods.”

#### 2.2.4. Sociodemographic Variables

Participants were asked their Hispanic/Latino background (Central American, Cuban, Dominican, Mexican, Puerto Rican, South American, and other/mixed), date of birth, gender, household income, and educational attainment.

### 2.3. Statistical Analysis

Age-adjusted or age-/sex-adjusted prevalence of overweight and obesity was estimated to describe the target population by sex, sociodemographic, and immigration and acculturation characteristics using design-based estimates from survey linear regression models [13]. To facilitate comparisons to NHANES, we also report the prevalence of obesity age-standardized to the year 2010 US population by sex and Hispanic/Latino background. To study the association (odds ratio) of Hispanic/Latino background, sociodemographic, immigration history (place of birth, years living in mainland US), and acculturation variables (language use and social relations SASH subscales, dietary acculturation) with BMI categories, we used survey generalized logit regression models. To assess the effect modification by Hispanic/Latino background we included separately the interaction term between each acculturation measure and background. We first tested whether this association was confounded by field center because in HCHS/SOL they are highly collinear due to the fact that people with specific Hispanic/Latino backgrounds tend to concentrate in specific geographic areas (Dominicans are predominantly in the Bronx, Cubans in Miami, and San Diego site mostly has Mexicans). Specifically, we tested the effect of field center (3 df) at an inflated significance level of 0.15 to be more conservative since some of the effect for field center can be absorbed by the estimates for background resulting in loss of power. If field center was not significant at this threshold, then this was interpreted as no evidence in which the effect of Hispanic/Latino background is confounded by field center and the effect of background can be directly assessed. In contrast, if field center was found to be significant, then we cannot know whether identified differences are due to Hispanic/Latino background or field center, and further analyses were conducted to understand these differences. We created a 17-level nominal variable formed from the cross classification of field center and Hispanic/Latino background including only cells where the sample size for the cross classification was greater than 100. Thus, we included the interaction between Hispanic/Latino background and field center without cells with very low sample size. We then used this 17-level covariate to fit a model to calculate the OR between field centers by Hispanic/Latino background. Lastly, among those born outside the US, we studied the association (odds ratio) of age at immigration with BMI group. All analyses accounted for the complex survey design and sampling weights (which include nonresponse adjustment relative to the sampling frame) using SAS version 9.3 corresponding procedures and SAS-callable SUDAAN version 10.

## 3. Results

Sixty percent of the sample was females (*N* = 9747). The mean age was 45.8 ± 13.9 years with Central Americans being the youngest group (44.5 ± 13.4) and Cubans the oldest (48.9 ± 13.2); 2,660 participants were in the 18–29-year age group, 2,371 in the 30–39, 4,178 in the 40–49, 4,299 in the 50–59, and 2,758 in the 60–74 age group. The majority of participants were first generation immigrants (81.0%) and of low socioeconomic status (38.2% did not graduate from high school, and 48.2% had an annual household income of <$20,000).

### 3.1. Prevalence of Overweight and Obesity

**Figure 1:**
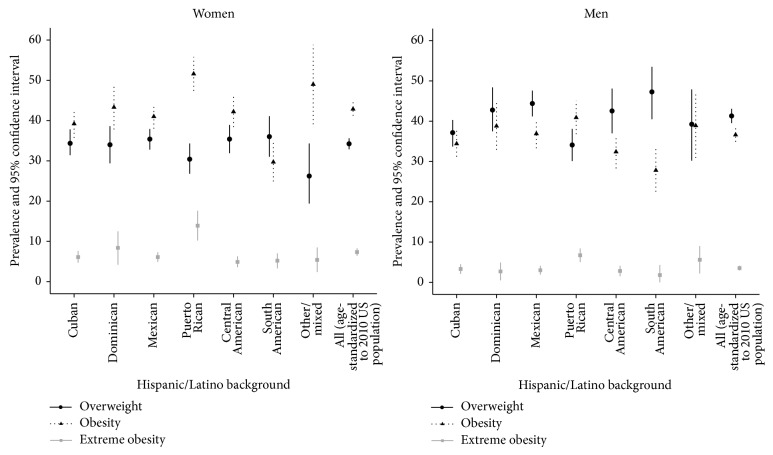
Age adjusted prevalence of overweight (BMI = 25–29 kg/m^2^), obesity (BMI ≥ 30 kg/m^2^), and extreme obesity (BMI ≥ 40 kg/m^2^) by sex and Hispanic/Latino background.

The overall age-adjusted prevalence of obesity (BMI ≥ 30 kg/m^2^) was 42.4% (95% CI 40.7, 44.0) for women and 36.5% for men (95% CI 34.7, 38.3). The overall age-adjusted prevalence of extreme obesity (BMI ≥ 40 kg/m^2^) was 7.4% in women and 3.7% in men. There appeared to be differences in the prevalence of obesity. The Bronx presented the largest prevalence of obesity (44.7%; 95% CI 42.1–47.2), followed by Chicago (42.1%; 95% CI 39.9, 44.4), San Diego (38.6%; 95% CI 35.4, 41.7), and Miami (36.7%; 95% CI 34.9, 38.6). However, some of the confidence intervals overlapped. Differences in extreme obesity followed the same pattern; the Bronx had the highest prevalence of extreme obesity (7.1%; 95% CI 5.6, 8.6), followed by Chicago (5.5%; 95% CI 4.7, 6.4), San Diego (5.0%; 95% CI 3.8, 6.2), and Miami (4.2%; 95% CI 3.4, 5.0). The age-adjusted prevalence of obesity varied across Hispanic/Latino backgrounds, with the highest prevalence observed among women and men of Puerto Rican background and the lowest among individuals of South American background (Figure 1). Women and men of Puerto Rican background also had the highest prevalence of extreme obesity, with almost double the prevalence rates seen in their counterparts.

### 3.2. The Association of Sociodemographic and Acculturation Measures with Overweight and Obesity

Table 1 presents results from the generalized logit model that examined whether the differences in BMI categories were due to differences across Hispanic/Latino backgrounds or to differences across field centers. Individuals of Puerto Rican background living in the Bronx were more likely to have extreme obesity, compared to Puerto Ricans living in Chicago, but moderate obesity was similar in these two groups. Individuals of Mexican background living in San Diego were less likely to have moderate obesity than those living in the Bronx but were more likely to have extreme obesity. Individuals of South American background in the Bronx were more likely to be overweight and have moderate obesity than those living in Miami. Among individuals of Central American background, obesity did not vary across field centers.

**Figure 2:**
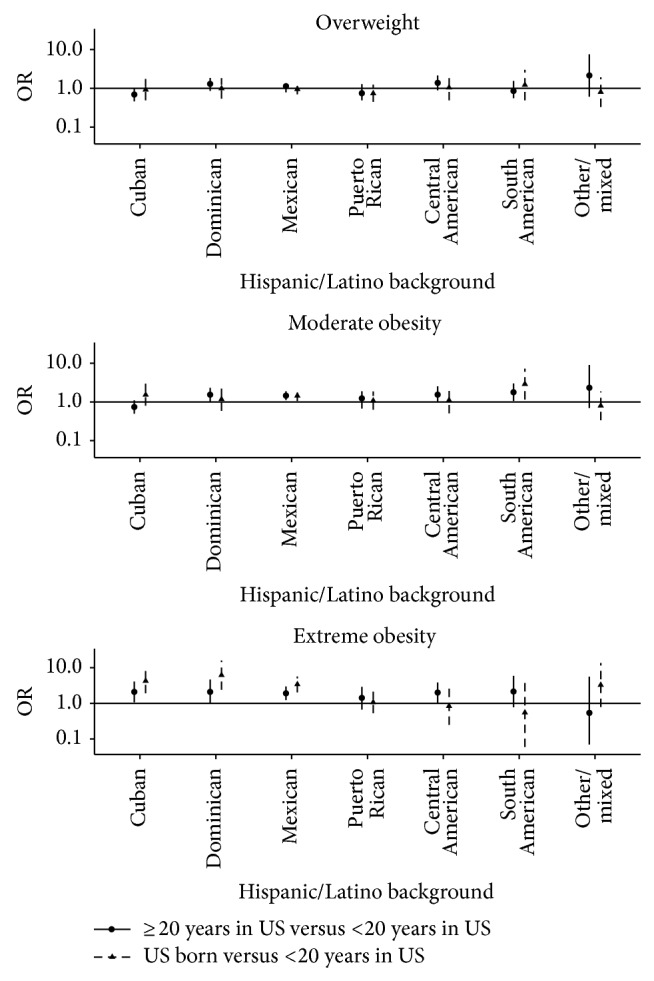
Odds ratio (OR) for the association of years living in the US with overweight (BMI = 25–29.9 kg/m^2^), moderate obesity (BMI = 30–39 kg/m^2^), and extreme obesity (BMI ≥ 40 kg/m^2^) by Hispanic/Latino background. Models are adjusted for age, sex, household income, educational attainment, acculturation field center, and sampling weights. Reference category is living in the US for <20 years.

Household income and educational attainment were not associated with moderate obesity. However, individuals with annual household income <$40,000 were more likely to have extreme obesity compared to participants with annual income >$40,000. US born participants and those living in the US for 20 or more years were more likely to have moderate obesity than those who had moved to the US less than 20 years ago. This association was much stronger for extreme obesity (Table 1) and was consistent across all Hispanic/Latino background groups (Figure 2). SASH language or the SASH social relations subscales were not associated with moderate or extreme obesity. However, higher social acculturation was inversely associated with overweight (Table 1). Dietary acculturation was not related to overweight or moderate obesity. However, individuals reporting eating Hispanic or American foods in equal amounts were more likely to have extreme obesity, compared to individuals eating mostly Hispanic foods.

Among the foreign-born, individuals who moved to the US when they were younger than 14 years old were more likely to have moderate (OR = 1.5; 95% CI 1.1, 1.9) and extreme obesity (OR = 2.2; 95% CI 1.4, 3. 5) than individuals immigrating at an older age; these results were adjusted for age, sex, Hispanic/Latino background, field center, household income, and educational attainment.

## 4. Discussion

The HCHS/SOL study found a high prevalence of obesity among adults aged 18–74 years. The age-standardized prevalence rates of obesity (42.9% in women and 36.6% in men) and extreme obesity (7.3% in women and 3.5% in men) were similar to estimates reported by NHANES 2009-2010 for Hispanic women (41.4% obesity and 6% extreme obesity) and men (37% obesity and 4.1% extreme obesity) [1]. The obesity prevalence for Puerto Ricans in HCHS/SOL exceeded these national estimates. Furthermore, the prevalence of extreme obesity among women and men of Puerto Rican background was almost twice the national estimates. This is particularly alarming as extreme obesity is associated with increased morbidity and mortality [8]. Although women and men of South American background had the lowest rates of obesity, the prevalence of overweight among them was comparable to the rates of overweight in the other Hispanic/Latino groups. These findings confirm that obesity is a major public health problem in the US Hispanic/Latino population and has implications for future health care costs given the association between obesity and increased risk of diabetes and cardiovascular disease [11, 14].

The study also showed that the prevalence of obesity and extreme obesity varied by field center and that some of the differences in Hispanic/Latino backgrounds could be explained by differences in field centers. Hispanic/Latino background differences were similar across field centers, except for the Bronx. Puerto Ricans living in the Bronx were more likely to have extreme obesity than Puerto Ricans from Chicago. South Americans from the Bronx were more likely to be overweight and have moderate obesity than those from Miami. Individuals of Mexican background in the Bronx were more likely to have moderate obesity but less likely to have extreme obesity, when compared to Mexicans from San Diego. These findings suggest that, at least for some Hispanic/Latino groups, place matters in terms of obesity risk. For example, the Bronx is one of the poorest counties in the state of New York, ranking high in food insecurity in national surveys [15]; low density of food stores and high proportion of unhealthy food outlets also have been reported for the Bronx [16]. Thus, the social and built environment characteristics of the Bronx may explain why certain groups have a higher burden of moderate and extreme obesity.

Consistent with previous studies, we observed that Hispanics/Latinos who were born in the US [4, 17], had lived longer in the US [18–23], or arrived in the US at an early age had the highest prevalence of obesity [20] and extreme obesity. These findings are also consistent with literature involving non-Hispanic immigrants [24]. The high prevalence of obesity among Hispanics/Latinos who have been living in the US for more than 20 years raises questions about the social and environmental conditions impacting risk. These findings suggest that prolonged exposure to the obesogenic US environment, which is conducive of diets rich in energy dense foods and low physical activity, plays a greater role in explaining excess weight among Hispanics/Latinos than acculturation per se, a point that has been made by others [4, 25, 26]. It is possible that longer duration of residency allows for more time for social and environmental factors to influence changes in behaviors and risk of obesity [5]. It is also important to note that this pattern of more obesity with longer duration of residency in the US was consistent across Hispanic/Latino backgrounds; as such, it reinforces the notion that longer exposure to an obesogenic environment is widespread across the country. However, as these analyses were cross-sectional, we must interpret results with caution. This limitation may be relevant as the association of length of residency in the US with obesity may be due to secular trends of increasing weight affecting all segments of the population and not the duration of residency per se [27].

Among the foreign-born, those who had immigrated to the US before the age of 14 years were more likely to have moderate and extreme obesity compared to participants who moved to the US in their twenties or older. This information supports the growing body of evidence regarding the health risks faced by “Generation 1.5” [2], those who immigrate before the age of 14. More research is needed on how to prevent further escalation of weight and associated health consequences in this population.

This study did not find an association of obesity with acculturation, as measured by the SASH language and social relations subscales. The process of acculturation is complex, and there is as yet no consensus on the best approach for comprehensive assessments of acculturation in epidemiological studies [28–30]. HCHS/SOL used a short scale whose constructs assess language acculturation and ethnic social relations (social acculturation), but it did not capture biculturalism. It is possible that a more refined measure of acculturation that could identify people as bicultural or with different degrees of acculturation across other domains (e.g., exposure to media) could reveal different patterns of association. Dietary acculturation was not associated with overweight or moderate obesity, but individuals who reported eating equal amounts of Hispanic and American foods were more likely to have extreme obesity than individuals who reported eating mainly Hispanic foods. This finding is interesting and warrants further exploration, but it is also difficult to interpret given the simplicity of the measure used. Examining detailed dietary patterns to understand what dietary choices people make in the process of acculturation is an area that needs to be examined. Furthermore, examining the environmental characteristics of the communities in which these participants live may shed light on the processes that put minority and low socioeconomic status populations at risk for obesity. Limited access to healthy foods and safe places to exercise while living in socioeconomically deprived areas are possible explanations for excess weight gain among low income individuals [31]. In addition, recent studies suggest that living in communities that are ethnic enclaves or ethnically isolated may play an important role [32, 33], which merits further exploration as obesity may spread through social networks [34]. The extent to which these social and environmental factors are relevant for acculturation research in Hispanics remains to be examined. Future longitudinal analyses of the HCHS/SOL data will contribute important information on the causal relationships between health behaviors and obesity risk, as well as the extent to which immigration and acculturation variables moderate this relationship.

## Figures and Tables

**Table 1:** Adjusted odds ratio (OR)^∗^ for the association of moderate and extreme obesity with Hispanic background, sociodemographic variables, and acculturation.

	Overweight (BMI = 25–29.9 kg/m^2^) OR (95% CI)	Moderate obesity (BMI = 30–39 kg/m^2^) OR (95% CI)	Extreme obesity (BMI ≥ 40 kg/m^2^) OR (95% CI)
Hispanic/Latino background			
Central-American			
Bronx versus Chicago	1.07 (0.56, 2.04)	1.40 (0.76, 2.59)	1.06 (0.40, 2.81)
Miami versus Chicago	0.99 (0.64, 1.54)	1.33 (0.92, 1.93)	1.52 (0.75, 3.07)
Bronx versus Miami	1.07 (0.58, 1.98)	1.05 (0.60, 1.85)	0.70 (0.29, 1.69)
Mexican			
Bronx versus Chicago	1.42 (0.83, 2.43)	1.66 (0.94, 2.93)	0.13 (0.03, 0.58)
Chicago versus San Diego	1.06 (0.86, 1.31)	1.30 (1.02, 1.64)	1.40 (0.95, 2.07)
San Diego versus Bronx	0.66 (0.38, 1.15)	0.47 (0.26, 0.83)	5.48 (1.23, 24.50)
Puerto Rican			
Bronx versus Chicago	1.39 (0.97, 2.01)	1.22 (0.83, 1.80)	1.78 (1.04, 3.06)
South American			
Bronx versus Chicago	1.31 (0.67, 2.57)	1.32 (0.63, 2.76)	2.49 (0.78, 7.90)
Chicago versus Miami	1.60 (1.02, 2.51)	1.62 (0.96, 2.75)	0.66 (0.19, 2.22)
Bronx versus Miami	2.10 (1.05, 4.18)	2.15 (1.06, 4.37)	1.63 (0.51, 5.26)
Household income			
Missing	0.77 (0.60, 0.99)	0.81 (0.62, 1.06)	0.82 (0.53, 1.29)
≤$20,000	1.05 (0.87, 1.28)	1.13 (0.92, 1.38)	1.62 (1.17, 2.26)
$20,001–$40,000	0.99 (0.81, 1.21)	1.10 (0.89, 1.36)	1.65 (1.16, 2.34)
>$40,000	Reference	Reference	Reference
Educational attainment			
Less than high school education	1.10 (0.93, 1.32)	1.19 (1.00, 1.42)	1.21 (0.90, 1.62)
High school education or equivalent	1.07 (0.91, 1.25)	1.10 (0.93, 1.31)	1.30 (0.95, 1.77)
Greater than high school education	Reference	Reference	Reference
Number of years living in the US			
Less than 20 years	Reference	Reference	Reference
≥20 years	0.98 (0.82, 1.17)	1.30 (1.10, 1.54)	2.12 (1.59, 2.83)
Born mainland US	0.91 (0.72, 1.16)	1.30 (1.00, 1.69)	2.77 (1.88, 4.08)
Acculturation (SASH)			
Language acculturation	1.03 (0.94, 1.14)	1.06 (0.94, 1.18)	1.16 (1.00, 1.35)
Social relations	0.87 (0.77, 0.99)	0.91 (0.80, 1.05)	0.95 (0.78, 1.17)
Type of food eaten (dietary acculturation)			
Mainly Hispanic	Reference	Reference	Reference
Mostly Hispanic	1.05 (0.91, 1.23)	0.96 (0.83, 1.10)	1.22 (0.93, 1.60)
Equal amounts	1.03 (0.84, 1.27)	1.07 (0.89, 1.30)	1.45 (1.04, 2.02)
Mainly/mostly American	0.88 (0.61, 1.27)	1.03 (0.71, 1.50)	0.55 (0.31, 0.95)

^∗^From generalized logistic regression model adjusted for all covariates listed in the table plus sex, age group, and sampling weights.
